# A case report of ventricular fibrillation following Shockwave intravascular lithotripsy during percutaneous coronary intervention

**DOI:** 10.1186/s12872-024-03894-z

**Published:** 2024-04-23

**Authors:** Lars Jakobsen, Evald Høj Christiansen, Troels Thim

**Affiliations:** Department of Cardiology, Aarhus University Hospital, Palle Juul-Jensens Boulevard 99, Aarhus, DK-8200 Denmark

**Keywords:** Ventricular fibrillation, Percutaneous coronary intervention, Complication

## Abstract

**Background:**

Shockwave intravascular lithotripsy (S-IVL) is widely used during percutaneous coronary intervention (PCI) of calcified coronary arteries. Ventricular capture beats during S-IVL are common but arrhythmias are rare.

**Case presentation:**

A 75-year-old woman was scheduled for PCI to a short, heavily calcified chronic total occlusion of the right coronary artery. After wiring of the occlusion, S-IVL was used to predilated the calcified stenosis. During S-IVL, the patient developed ventricular fibrillation twice.

**Conclusion:**

To our knowledge, this is only the second reported case of VF during S-IVL. Although very rare, it is important to be aware of this potential and serious complication.

## Introduction

Shockwave intravascular lithotripsy (S-IVL) is considered to facilitate successful stent implantation in severely calcified coronary lesions with a high rate of procedural success and low risk of complications [1]. It is well known that S-IVL can cause ventricular capture beats during treatment [2]. However, arrhythmias caused by S-IVL are very rare. A pooled analysis of the Disrupt CAD I-IV reported no arrhythmias and only few cases of supraventricular arrhythmias have been reported [3, 4]. To the best of our knowledge, there are no reports of ventricular tachycardia and only one report of ventricular fibrillation (VF) caused by S-IVL [5]. In this case report, we present a detailed description of a patient who experienced VF during S-IVL.

## Case report

A 75-year-old woman was scheduled for percutaneous coronary intervention (PCI) to a chronic total occlusion (CTO) on the right coronary artery (RCA). The patient was known with hypertension and dyslipidemia. She had impaired renal function with an estimated glomerular filtration rate of 35 ml/min/1.73 m^2^. The patient had a transient ischemic attack in 2018 and was diagnosed with paroxysmal atrial fibrillation in 2020 after which she was started on direct oral anticoagulation. In 2020 she was diagnosed with sarcoidosis with involvement of mediastinal lymph nodes. There were no signs of pulmonary involvement.

In 2021 she was diagnosed with ischemic heart disease. A coronary angiogram showed 3-vessel disease including a CTO on the RCA. Left ventricular ejection fraction was 40%. Coronary artery bypass graft surgery was planned. However, besides the left internal mammary artery she had no vessels suitable for bypass grafts. Thus, only the left anterior descending artery was grafted. In March 2023, she was treated with PCI of the left main and circumflex artery because of symptoms in spite of optimal medical treatment.

**Fig. 1:**
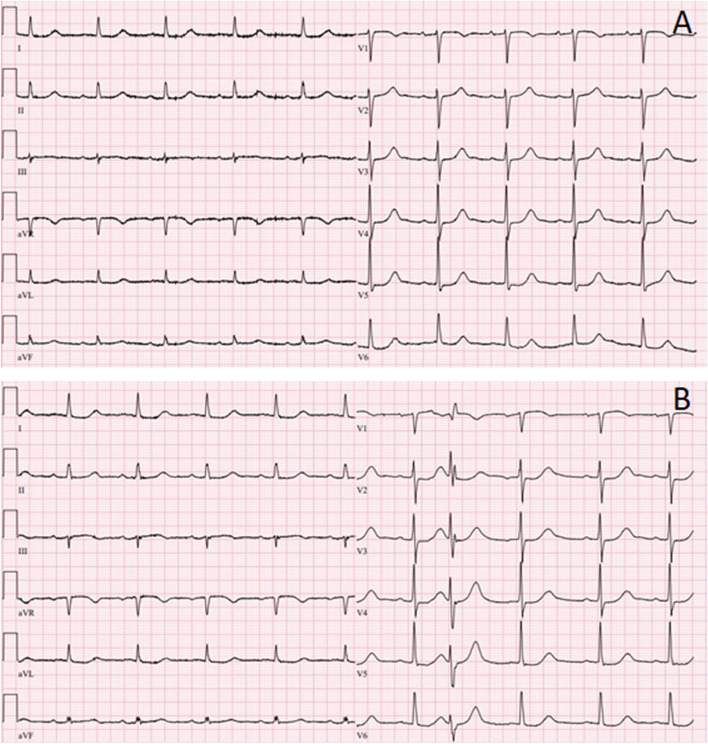
Pre- and post-procedure ECG. **A** Pre procedure and discharge electrocardiogram showing sinus rhythm and long QT interval. **B** Post procedure electrocardiogram showing sinus rhythm and very long QT interval

A O-15-H_2_O positron emission tomography scan showed severe hypo-perfusion in the RCA territory. With a Seattle Angina Questionnaire Quality of Life score of 50 out of 100, she fulfilled the criteria for inclusion in the Ischemia CTO trial and she was randomized to PCI of the RCA CTO [6]. Electrocardiogram (ECG) showed sinus rhythm with a long QT interval (QTc corrected by Bazett's and Fridericia's formula of 521 ms and 495 ms, respectively) which was known for several years (Fig. 1A). The patient was not on any medications known to cause QT interval prolongation.

**Fig. 2:**
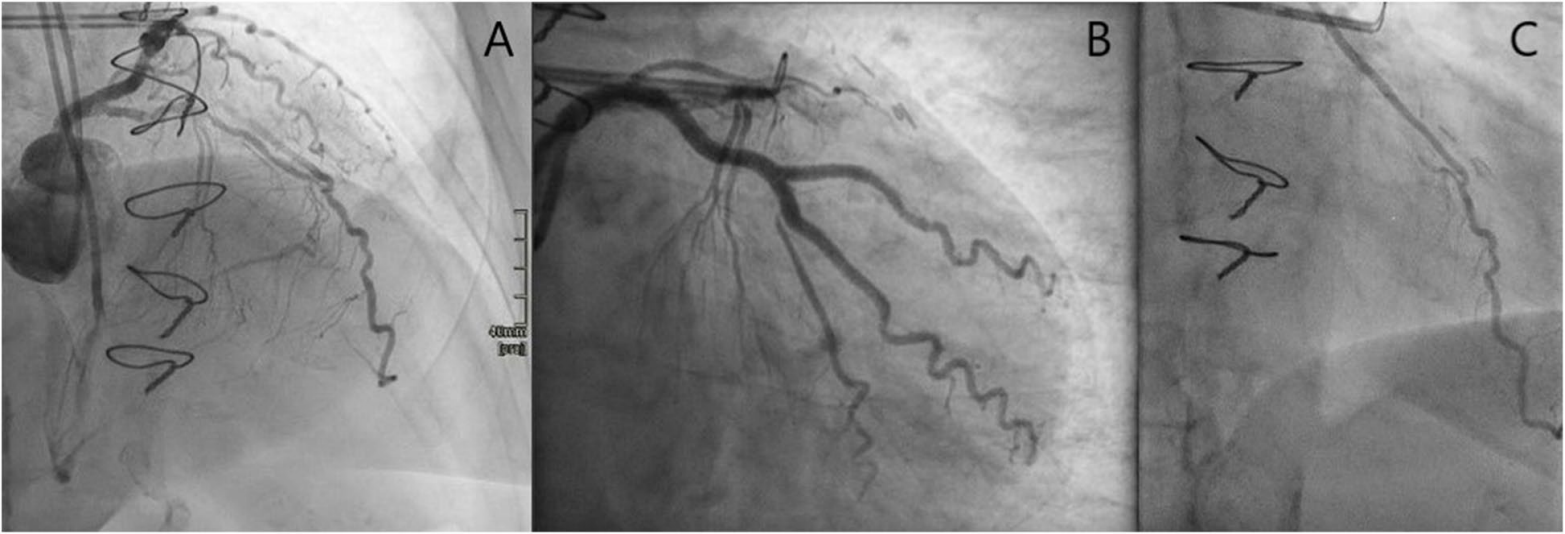
Coronary angiogram of the left coronary artery and left internal mammary artery to the left anterior descending artery. **A** Cranial view of the left anterior descending artery with a severe proximal stenosis. The distal part of left internal mammary artery to the left anterior descending artery is visible. **B** Caudal view of the left main and circumflex artery showing a good stent result. **C** The distal part of the left internal mammary artery and the anastomosis with the left anterior descending artery

**Fig. 3:**
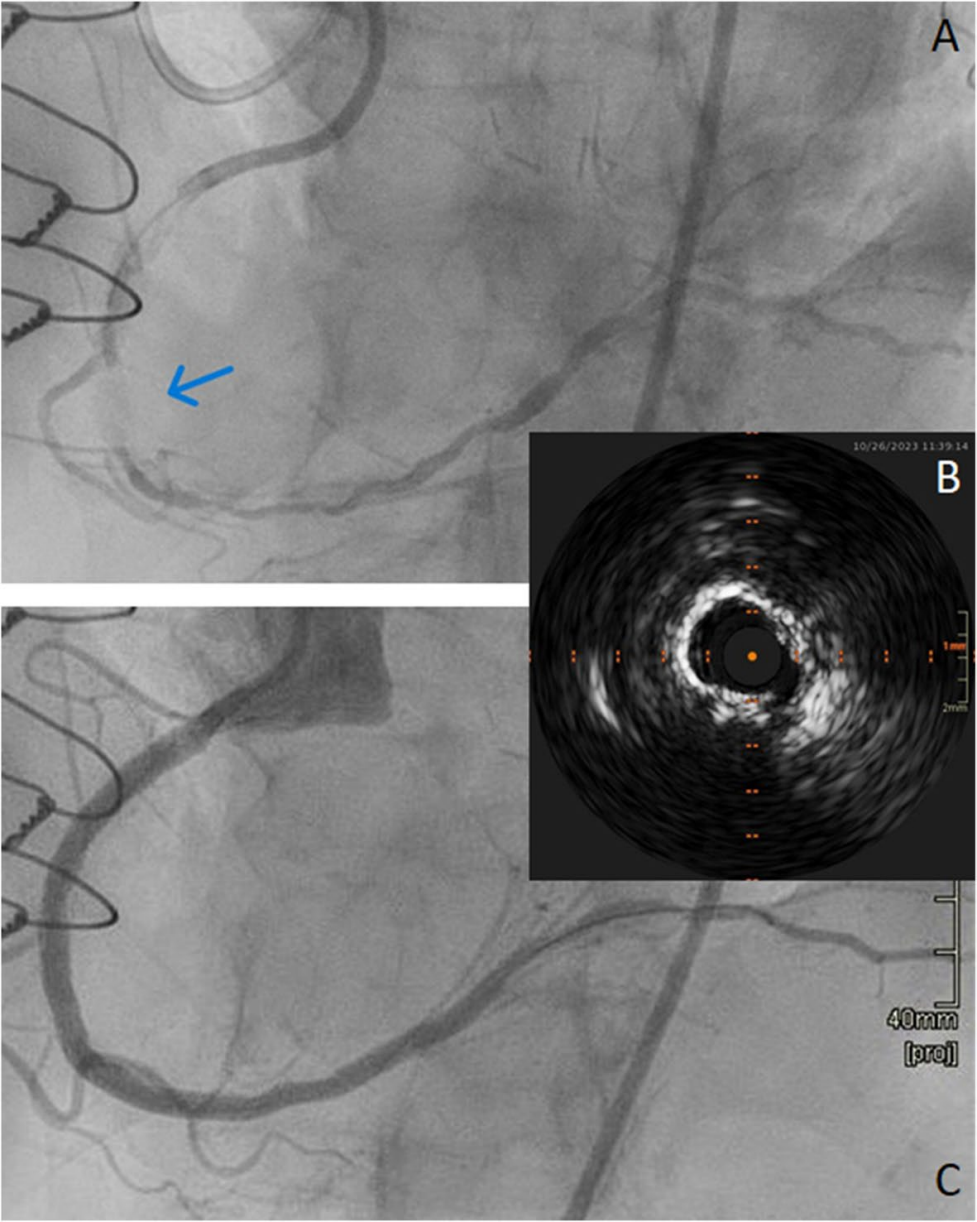
Coronary angiography and intravascular ultrasound image of the right coronary artery. **A** Right coronary artery chronic total occlusion (blue arrow) before treatment with Shockwave Intravascular Lithotripsy and percutaneous coronary intervention. **B** Intravascular imaging of the right coronary artery showing circular calcification. **C** Final result after percutaneous coronary intervention of the right coronary artery

**Fig. 4:**
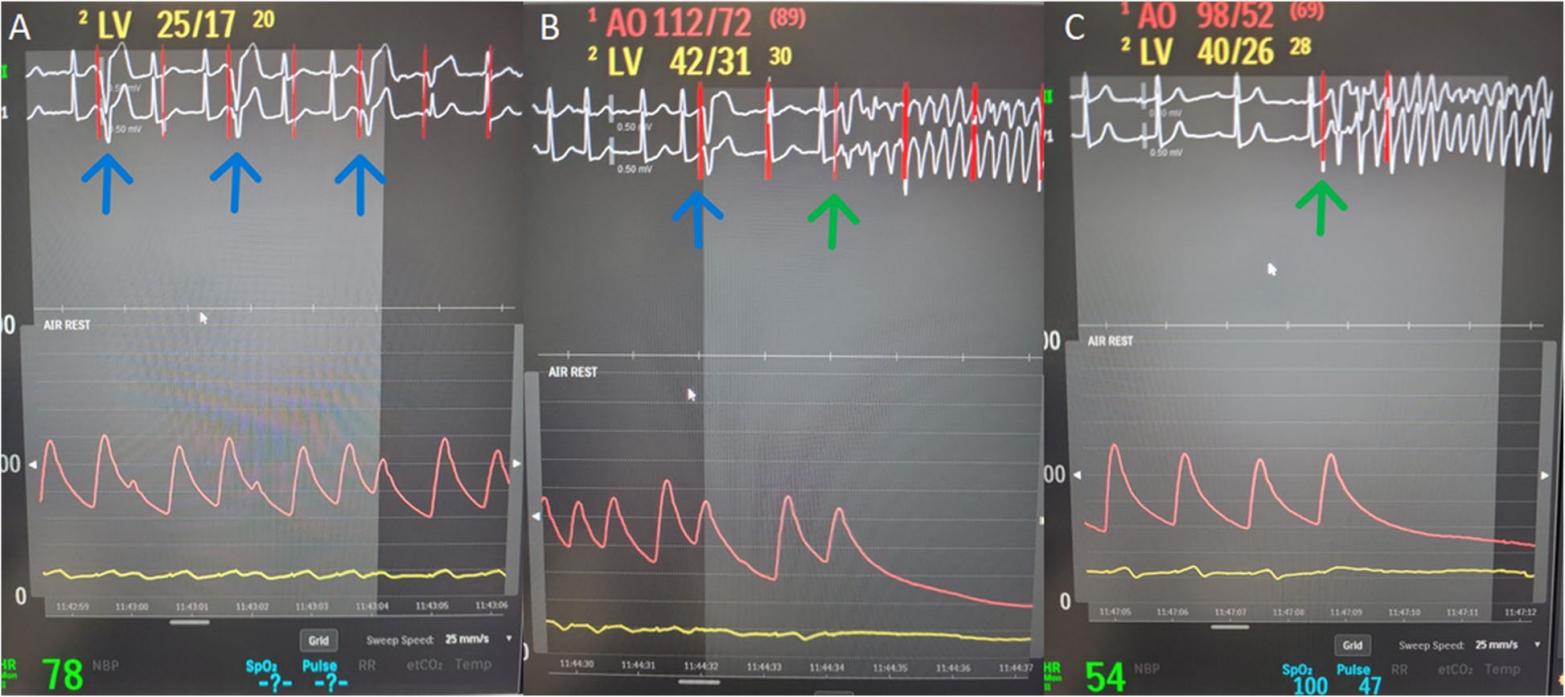
Heart rhythm monitoring during Shockwave Intravascular Lithotripsy. **A** Capture beats (blue arrows) caused by Shockwave Intravascular Lithotripsy. **B** Capture beat (blue arrow) and superimposition of a ventricular capture beat on the T wave of the preceding beat (green arrow) followed by ventricular fibrillation. **C** Superimposition of a ventricular capture beat on the T wave of the preceding beat (green arrow) followed by ventricular fibrillation

A coronary angiography showed a severe mid-LAD stenosis, a good stent result in the left main and circumflex artery and a good left internal mammary artery to the LAD (Fig. 2A to C). A dual coronary angiogram showed a short heavily calcified mid-RCA CTO (Fig. 3A) and long segments proximal and distal to the CTO with significant disease. The occlusion was passed with retrograde wiring. The RCA was predilated with a 2.0 × 20 mm compliant balloon followed by a 2.5 × 15 mm non-compliant balloon before imaging with intravascular ultrasound. Intravascular ultrasound showed areas with circular calcification (Fig. 3B) throughout the diseased segment of the RCA. Thus, S-IVL with a 3.5 × 12 mm Shockwave balloon was planned. The first 70 impulses were delivered between the distal landing zone and just proximal to the CTO. After additional 5 impulses, the patient suddenly had VF and cardiac arrest. She was immediately defibrillated to sinus rhythm and recovered completely in less than two minutes. It was thought to be unlikely that VF was caused by the S-IVL. Thus, additional S-IVL was performed. However, after the very first of the subsequent impulses was given she had VF and cardiac arrest. Again, the patient was defibrillated and recovered. No further S-IVL impulses were given and the rest of the procedure was uneventful. The vessel was further predilated with NC-balloons and stented from the ostium to the posterior descending artery with a good final result (Fig. 3C). After the procedure, the ECGs recorded during the procedure were reviewed. There were several episodes with ventricular capture beats (Fig. 4A). It is also clear that both episodes of VF were initiated by the R-on-T phenomenon caused by the superimposition of a ventricular capture beat on the T wave of the preceding beat (Fig. 4B and C). During and after the procedure, the patient´s heart rate was around 60 beats per minute (bpm) which was lower than her normal heart rate of 80–90 bpm. Post procedure ECG showed sinus rhythm, 60 bpm and a very long QT interval, QTc corrected by Bazett's and Fridericia's formula of 534 ms and 537 ms, respectively (Fig. 1B). Blood test showed normal electrolytes including normal plasma potassium, magnesium and calcium.

Because of the post procedure ECG with very long QT interval, she stayed in hospital overnight for heart rhythm monitoring. There were no further arrhythmias. She was discharged the next day. The ECG at discharge was identical to the pre procedure ECG (Fig. 1A).

## Discussion

A patient-level pooled analysis of the Disrupt CAD I-IV Studies including 628 patients reported no arrhythmias [1]. One case of self-limiting atrial fibrillation caused by capture beats during S-IVL to the proximal RCA has been reported [3]. Furthermore, one case of atrial flutter during S-IVL to the proximal left anterior descending artery has been reported [4]. The Disrupt CAD III Study included 431 patients treated with S-IVL [2]. Heart rhythm assessment was evaluable in 416 patients. IVL-induced capture beats were seen during S-IVL in 41.1% of cases. S-IVL-induced capture beats did not result in sustained ventricular tachycardia or VF during or immediately after the procedure in any patient and were not associated with adverse events. Cox regression analysis identified heart rate ≤ 60 bpm, male sex, and total number of S-IVL pulses delivered as independent predictors of S-IVL-induced capture beats. To the best of our knowledge, VF caused by S-IVL has only been reported once previously [5]. In that case report, a spontaneous ventricular ectopic beat was followed by a compensatory pause before the next sinus beat and then a S-IVL capture beat during repolarization causing VF. The patient’s spontaneous ventricular ectopic beat caused a short–long–short sequence which may act as one susceptibility-substrate for the S-IVL triggered VF. The patient in the present case had sinus bradycardia during the procedure which, according to the data presented above, increases the risk of capture beats. Furthermore, the patient's ECG showed a long QT interval, especially in the ECG taken immediately after the procedure. The long QT interval makes the heart more vulnerable to VF which is frequently preceded by one or more premature ventricular beats coupled to the prolonged QT segment of the preceding basic beat [7]. The combination of S-IVL, sinus bradycardia and long QT interval might explain the episodes with VF. The R-on-T phenomenon occurs when a ventricular premature (spontaneous or paced) complex falls during a vulnerable period of repolarization. If the R-on-T phenomenon is caused by a pacemaker, or as in this case S-IVL, it is often related to ventricular under sensing problems or asynchronous pacing [8]. The S-IVL causes asynchronous pacing when the S-IVL results in capture beats. Thus, the potential life threatening complication described in the present case could potentially be avoided by electrocardiography synchronized S-IVL to avoid causing capture beats in the vulnerable period of the cardiac cycle. The most common cause of arrhythmias, including VF, during coronary intervention procedures is ischemia. Also in this case, ischemia is a potential alternative trigger of VF.

## Conclusion

We report a case of VF and cardiac arrest associated with S-IVL. Although very rare, it is important to be aware of this potential and serious complication.

## Data Availability

Data sharing is not applicable to this article as no datasets were generated or analysed during the current study.

## Abbreviations

BPM: Beats per minute
CTO: Chronic total occlusion
ECG: Electrocardiogram
PCI: Percutaneous coronary intervention
RCA: Right coronary artery
S-IVL: Shockwave Intravascular Lithotripsy
VF: Ventricular fibrillation
